# Vanishing Bile Duct Syndrome Associated With Non-Hodgkin’s Lymphoma and Hepatitis E Virus Infection

**DOI:** 10.7759/cureus.21328

**Published:** 2022-01-17

**Authors:** Mansoor Zafar, Mariya Farooq, William Butler- Manuel, Mohammad Fawad Khattak, Usman Iqbal Rana, Tila Muhammad, Ian Hawley, Mark Whitehead, Muhammad Toqeer

**Affiliations:** 1 Gastroenterology and Hepatology, Conquest Hospital, East Sussex Healthcare NHS Trust, St. Leonards-on-Sea, GBR; 2 General Internal Medicine, Conquest Hospital, East Sussex Healthcare NHS Trust, St. Leonards-on-Sea, GBR; 3 Internal Medicine, Conquest Hospital, East Sussex Healthcare NHS Trust, St. Leonards-on-Sea, GBR; 4 Histopathology, Conquest Hospital, East Sussex Healthcare NHS Trust, St. Leonards-on-Sea, GBR; 5 Gastroenterology, Conquest Hospital, East Sussex Healthcare NHS Trust, St. Leonards-on-Sea, GBR

**Keywords:** non-invasive liver screen, deranged liver function tests, vanishing bile duct syndrome, hodgkin's lymphoma non-hodgkin's lymphoma, hepatis e virus

## Abstract

The vanishing bile duct syndrome (VBDS) is a condition secondary to inciting triggers resulting in destruction and eventual disappearance of intrahepatic bile ducts leading to cholestasis. The overall outcome varies and often depends on the nature of the precipitating cause. VBDS has been found to be associated with adverse drug reactions, infectious diseases, autoimmune diseases, ischemia, and humoral factors associated with malignancies and is often irreversible. The objective of this clinical case report is to highlight the need for a broad differential to include VBDS in similar scenarios to aid rapid diagnosis and management. We hope this could lead to a more favourable outcome for patients presenting with VBDS such as the one described in this case report with concurrent non-Hodgkin’s lymphoma and infection with hepatitis E virus. To the best of our knowledge, this is the first ever reported case of VBDS associated with non-Hodgkin’s lymphoma and hepatitis E virus infection.

## Introduction

The vanishing bile duct syndrome (VBDS) is an acquired disorder associated with progressive destruction of intrahepatic bile ducts with resultant cholestasis [1]. VBDS has been shown to be a sequela of autoimmune diseases including primary sclerosing cholangitis and primary biliary cholangitis. It can also be a sequela of the human immunodeficiency virus (HIV), cytomegalovirus, and Epstein-Barr virus (EBV). It can be associated with commonly prescribed antibiotics including amoxicillin, levofloxacin, meropenem, azithromycin, and sulfamethoxazole-trimethoprim, and antiepileptics such as carbamazepine, lamotrigine, and valproic acid. It is also linked to lymphomas including Hodgkin's, Non-Hodgkin's, B-cell, and T-cell lymphomas [2,3]. Non-Hodgkin lymphoma (NHL)-related VBDS has been reported as a rare occurrence, resulting in mortality associated with liver failure [4].

Here we present a unique case of an 83-year-old man with non-Hodgkin’s lymphoma and co-infection with hepatitis E virus resulting in VBDS. Following aggressive investigation, he was managed medically with resolution of symptoms and normalisation of biochemistry. This is only the second reported case of VBDS associated with non-Hodgkin's lymphoma in the last 10 years with the other reported case not having as favourable an outcome as our patient [4].

## Case presentation

An 83-year-old man presented to the emergency department with concerns of painless jaundice, mild pruritus, and weight loss of 4 kg in eight weeks. There was no past history of increased alcohol intake, drug abuse, or jaundice. There was no family history of liver disease or haematological disorders. The only exception was a remote history of a previous cholecystectomy. Clinical examination demonstrated visible scleral and skin icterus, palpable mild lymphadenopathy along the anterior and posterior cervical chains, and mild splenomegaly. He had no demonstrable clinical signs of hepatic encephalopathy. On further discussion, he gave a one-month history of lethargy and described light coloured stools and dark coloured urine. His bilirubin on admission was 324 umol/L (0-21 umol/L), which rose to a maximum of 646 umol/L. His alkaline phosphatase (ALP) was 450 u/L (0-120 u/L), alanine transaminase (ALT) was 725 u/L (10-35 u/L), and tests for paracetamol and salicylates were negative. Additionally, he developed coagulopathy over the days following admission with an international normalised ratio (INR) of 1.6 (0.8-1.2), which corrected to 1.1 following three days of 10 mg intravenous vitamin K administration. His non-invasive complete liver screen along with viral serology for hepatitis A, B, C, and HIV was negative. Immunoglobulins studies were unremarkable with no light chains demonstrated. Auto-immune profile was negative and his lactic acid dehydrogenase (LDH) was normal at 217 U/L (100-250 U/L). Surprisingly, his serology tests did demonstrate evidence of acute hepatitis E virus, IgG and IgM reactive. Subsequently, his hepatitis E RNA was found to be 1800 IU/ml. These results were consistent with acute hepatitis E virus infection.

**Figure 1:**
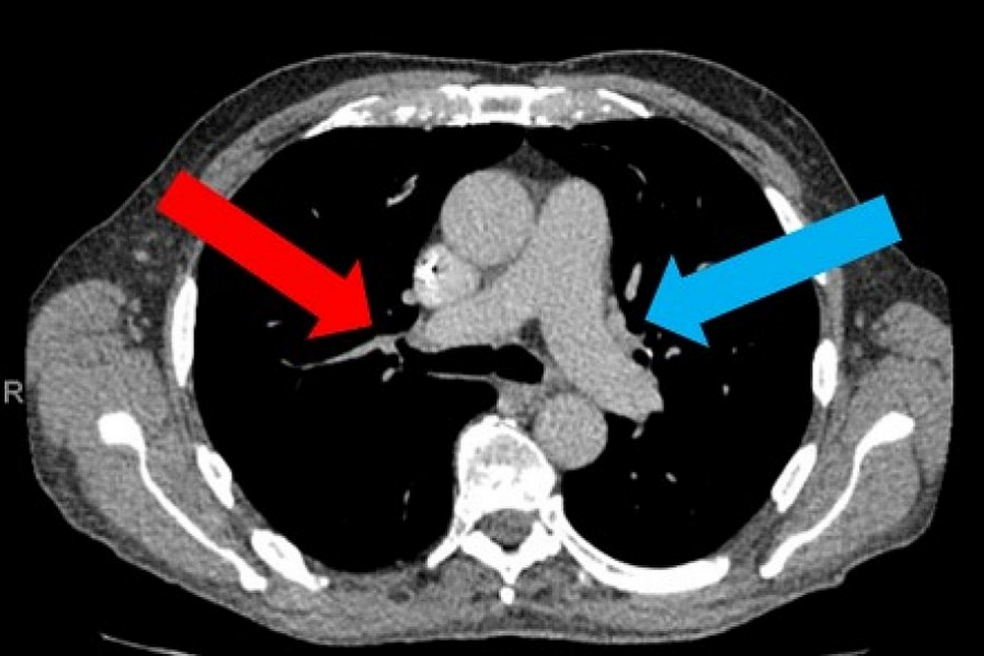
CT chest showing enlarged sub-aortic lymph nodes (blue arrow) and right lower paratracheal nodes (red arrow)

**Figure 2:**
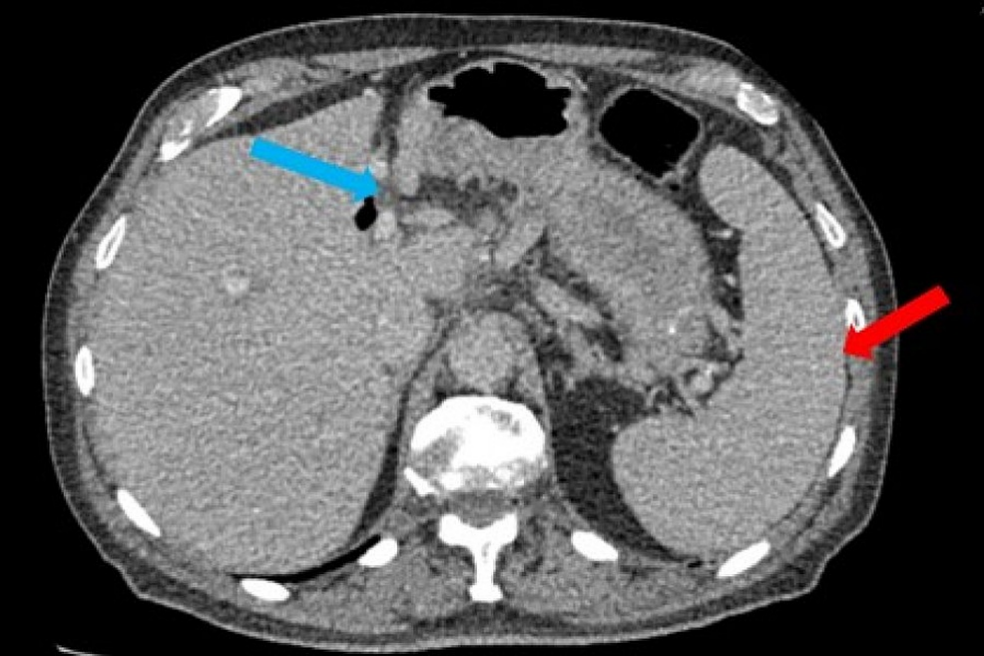
CT abdomen showing splenomegaly of 18.5 cm (red arrow) and multiple mesenteric lymph nodes (blue arrow)

A computed tomography (CT) scan showed no evidence of pancreatic abnormality as suspected; however, it showed the spleen to be mildly enlarged. Additionally, extensive para-aortic and mesenteric lymphadenopathy were seen. Some lymphadenopathy was also seen within the neck and chest (Figures 1-2).

**Figure 3:**
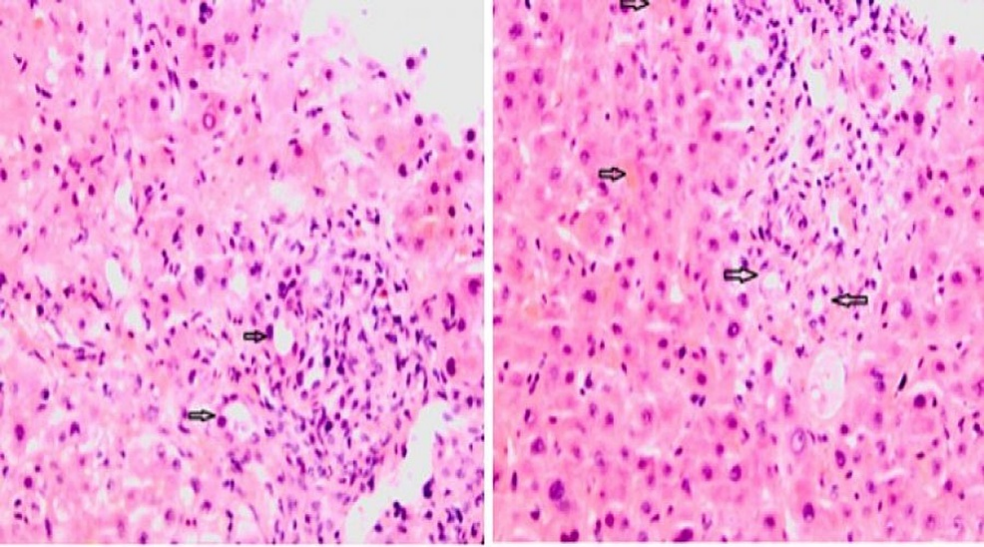
Liver biopsy: H&E staining, magnification x 200; (A) Damaged bile ducts in portal tract (black arrows), (B) Portal tract with damaged bile ducts and cholestasis (black arrows) H&E: hematoxylin and eosin

**Figure 4:**
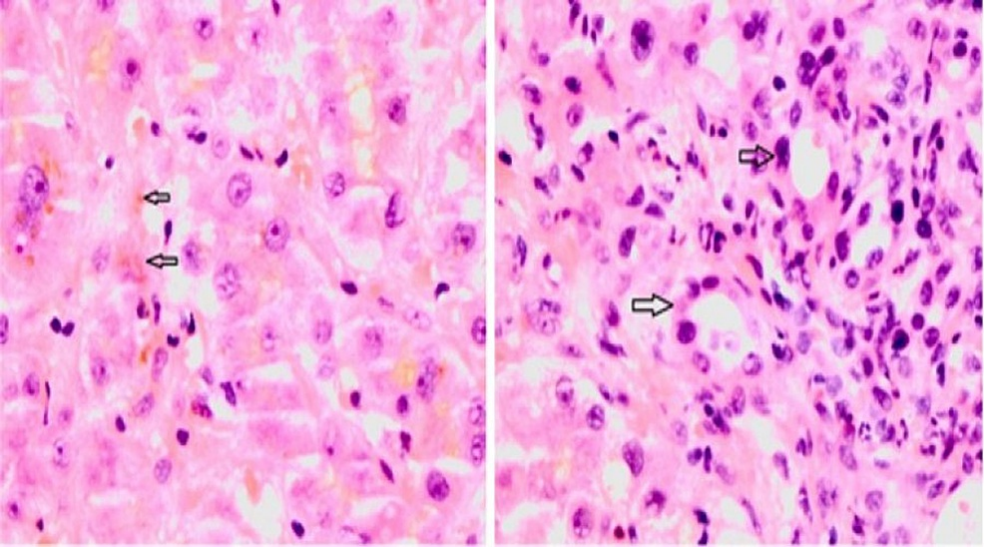
Liver biopsy: H&E staining, magnification x 400; (A) Canalicular and hepatocellular cholestasis (black arrows), (B) Damaged bile ducts in portal tract (black arrows) H&E: hematoxylin and eosin

His case was discussed in the multidisciplinary team meeting (MDM) and following discussion with the interventional radiology team, he underwent a liver biopsy that suggested features conclusive of VBDS (Figures 3-4).

No evidence of lymphoma was seen in the liver biopsy. The para-aortic lymph node biopsy demonstrated histological features of low-grade follicular non-Hodgkin's Lymphoma (grade 1/2). The bone marrow aspirate, trephine, and immunophenotyping did not show any evidence of infiltration by disease.

The clinical dilemma was the uncertainty around whether his VBDS was a paraneoplastic phenomenon in relation to his non-Hodgkin’s lymphoma or a reaction to his acute hepatitis E infection. As a result of his deranged liver function and acute infection, chemotherapy during his admission was not considered to be appropriate or safe. He was managed on steroid monotherapy with prednisolone 40 mg once a day along with a proton pump inhibitor (PPI) (Omeprazole 40 mg once a day) for gastroprotection and vitamin D-calcium supplement (Calcichew-D3 one tablet twice a day) for bone protection whilst on steroids. He was prescribed Chlorpheniramine Maleate (antihistamine) for pruritis as required with advice to refrain from driving due to the sedative effect of antihistamines. Following dietician review, a nasogastric (NG) tube was inserted for short time for nutritional supplementation along with periodic repeat refeeding blood tests including corrected calcium, phosphate, and magnesium. With satisfactory weight gain, the NG tube was eventually removed after 10 days.

One month post discharge, he was seen as an outpatient and his hepatitis E IgG and IgM were still reactive, but hepatitis E RNA was no longer detectable in his blood but remained detectable in his stool. His jaundice had continued to settle. Blood count and coagulopathy were unremarkable. His bilirubin had fallen to 56 umol/L. ALP was slightly elevated at 165 U/L and his ALT was normal. He was maintained on a weaning dose of steroids with a gradual reduction of 5 mg every week. Six months later, he underwent three cycles of chemotherapy with obinutuzumab, cyclophosphamide, vincristine, and prednisolone (O-CVP) under the haematology team and remains in remission. Three years later, he attends regular outpatient appointments with the haematology team along with periodic blood tests, including liver function tests, for surveillance.

## Discussion

VBDS was first described in 1993 by Hubscher et al. in association with Hodgkin’s lymphoma [5]. However, Ludwig et al. first described VBDS in 1988 as “idiopathic adulthood ductopenia” in a case series of three patients [6].

Intrahepatic cholestasis of pregnancy as a result of hormonal effect on pregnancy has occasionally been reported associated with VBDS. However, this usually resolves after the delivery of the foetus with no persistent increased bilirubin levels or ductopenia [7]. Multiple aetiologies have been implicated with VBDS as a sequela [8] including reaction to HIV treatment [8]. A case of VBDS has been reported to occur in a patient with AIDS, although it is queried if this was in association with CMV co-infection with low CD4 count [9].

The management of VBDS has been broad. Pruritis has been treated with antihistamines including diphenhydramine, hydroxyzine, and sedative antihistamines. More resistant pruritis has been reported managed with bile acid resins including cholestyramine and colestipol [10]. Corticosteroids have been used for severe cholestasis and VBDS [10]. Although there has been no prospective trial, anecdotal reports suggest the beneficial use of ursodiol for VBDS [10]. Calcineurin inhibitors and monoclonal antibodies have also been proposed [10]. However, a proportion of patients with VBDS eventually develop cirrhosis and end-stage liver disease, requiring liver transplantation [10]. It should also be considered that in some cases changes in liver function be attenuated by use of Urso-deoxycholic acid (UDCA) or by steroids [11].

To the best of our knowledge, we report the first-ever case of VBDS associated with non-Hodgkin’s lymphoma along with co-infection with Hepatitis E virus that responded to treatment with steroids with a comparatively favourable outcome. We encourage clinicians to consider VBDS as a differential diagnosis in cholestasis and encourage further case reports to aid data collection towards improving outcomes for VBDS, especially in cases with non-Hodgkin’s lymphoma and Hepatitis E virus co-infection.

## Conclusions

The phenomenon of VBDS requires early recognition followed by appropriate management. It is important to understand that delayed or reduced-intensity treatments lead to increased morbidity and mortality. VBDS can present via many pathways and remains an important differential with cholestatic liver function test results in patients with non-Hodgkin's lymphoma. Biopsy and histology are key to establishing the diagnosis. It should also be considered that in some cases, changes in liver function can be attenuated by steroids. Significant overlap exists in the clinical picture of VBDS versus obstructive jaundice secondary to the structural hepatic and post-hepatic aetiology. 
